# ICD-9 Codes for Identifying Influenza Hospitalizations in Children

**DOI:** 10.3201/eid1210.051525

**Published:** 2006-10

**Authors:** Ron Keren, Anna Wheeler, Susan E. Coffin, Theoklis Zaoutis, Richard Hodinka, Kateri Heydon

**Affiliations:** * The Children's Hospital of Philadelphia, Philadelphia, Pennsylvania, USA

**Keywords:** Influenza, surveillance, pediatrics, hospitalization, international classification of diseases, letter

**To the Editor:** The effect of influenza on young children is substantial, but most infections are clinically unrecognized (*1*). As a result, without routine laboratory confirmation of influenza infection in patients admitted to the hospital with influenzalike illness, accurate estimates of influenza-related hospitalization rates are difficult to obtain. Several statistical models have been developed to generate estimates of excess or influenza attributable hospitalizations, all of which calculate the rate of hospitalization above baseline during periods in which influenza is circulating (*2**–**8*). However, their accuracy is limited when viruses such as respiratory syncytial virus (RSV) and parainfluenza are cocirculating with influenza.

International Classification of Diseases 9th revision (ICD-9) diagnostic codes specific to influenza (487.0, 487.1, and 487.8) are easily retrieved from hospital discharge records. However, researchers and public health officials have rarely used them for influenza hospitalization surveillance, presumably because they lack sensitivity for identifying true influenza infections, although this assumption has never been tested.

To determine the sensitivity and positive predictive value of influenza-specific ICD-9 admission or discharge codes (487.0, 487.1, and 487.8), we conducted a retrospective cohort study of all patients <21 years of age hospitalized at the Children's Hospital of Philadelphia with laboratory-confirmed influenza during 3 consecutive influenza seasons (July 2001 through June 2004) (*9*). We compared admission and discharge ICD-9 codes with influenza laboratory results. All specimens were initially tested by rapid solid-phase immunoassay for RSV (Binax; Portland, ME, USA) and influenza (Binax). Direct fluorescent antibody testing for adenovirus, influenza A and B, parainfluenza virus types 1, 2, and 3, and RSV was performed on specimens negative by solid-phase immunoassay for RSV or influenza. Comprehensive viral culture was established for all specimens negative for respiratory viruses by direct fluorescent antibody test.

Of 715 cases of laboratory-confirmed influenza identified (Table), 617 (86%) were identified by rapid testing and 98 (14%) by viral culture after rapid test results were negative. A total of 529 patients had influenza-specific admission or discharge ICD-9 codes. The sensitivity of influenza-specific ICD-9 codes was 65% (95% confidence interval [CI] 61%–68%), and the positive predictive value was 88% (95% CI 84%–90%) (Table). Of 66 patients who had influenza-specific admission or discharge ICD-9 codes but negative influenza laboratory results, laboratory tests confirmed parainfluenza (n = 42), *Haemophilus influenzae* (n = 6; 1 with a positive blood culture and 5 with positive respiratory cultures), *H*. *parainfluenzae* (n = 1 wound infection), adenovirus (n = 1), and RSV (n = 2) infections. For 5 patients, influenza infection was documented in their charts, but they had either negative influenza test results or no influenza test performed. Seven patients had the expression "follow-up" written as "f/u" in the assessment section of their admission note, which may have been interpreted by medical coders as flu. We could not determine the reason for miscoding in 2 patients.

**Table:** Influenza-specific admission or discharge ICD-9 codes (487.0, 487.1, and 487.8.) compared with influenza laboratory test results*

Parameter	LCI	No LCI	Total
Influenza-specific diagnosis codes	463	66	529
No influenza-specific diagnosis codes	252	–	–
Total	715	–	–

*ICD-9, International Classification of Diseases-9; LCI, laboratory-confirmed influenza. The sensitivity and positive predictive value of influenza-specific diagnosis codes were 65% and 88%, respectively.

The sensitivity of influenza-specific diagnosis codes was related to the method of laboratory confirmation. Seventy-three percent (452/617) of patients (95% CI 70%–77%) who had positive rapid test results had influenza-specific admission or discharge diagnosis codes, whereas only 11% (11/98) (95% CI 6%–19%) who had positive influenza viral cultures (and negative rapid test results) had influenza-specific diagnosis codes.

Our results have a few policy implications. First, they suggest that in hospitals where routine influenza viral testing is performed, use of admission and discharge ICD-9 codes from hospital billing data for surveillance purposes will systematically underestimate actual influenza-related hospitalizations by 35%. The higher sensitivity of influenza-specific ICD-9 codes in patients with positive rapid test results compared with positive culture results suggests that unlike viral culture results, which generally are not available before discharge, rapid test results are often used to assign influenza-specific ICD-9 codes. Thus, rapid diagnostic tests that are more sensitive (e.g., PCR-based assays) may increase the sensitivity of influenza-specific ICD-9 codes in hospitals that routinely evaluate children admitted with respiratory symptoms of unclear cause. However, the imperfect specificity (94%–98%) of rapid influenza tests will produce a small but not negligible number of false-positive results. In hospitals where influenza testing is not commonly performed, the sensitivity of influenza-specific ICD-9 codes is likely to be lower.

Second, the high positive predictive value of influenza-specific ICD-9 codes observed in this study suggests that in hospitals where influenza testing is routinely performed, most patients whose hospitalization summary includes an influenza-specific ICD-9 code actually have influenza. However, misclassification of patients with parainfluenza and *H*. *influenzae* infections as patients with influenza demonstrates the potential for systematic coding errors even when influenza testing is routine.

Epidemiologists and public health officials should be aware that influenza-specific ICD-9 codes assigned in a setting of routine rapid diagnostic testing may be useful for following trends. However, these codes will substantially underestimate the actual number of influenza-related hospitalizations.
